# Prevalence of welfare outcomes in the weaner and finisher stages of the production cycle on 31 Irish pig farms

**DOI:** 10.1186/s13620-018-0121-5

**Published:** 2018-03-27

**Authors:** Nienke van Staaveren, Julia Adriana Calderón Díaz, Edgar Garcia Manzanilla, Alison Hanlon, Laura Ann Boyle

**Affiliations:** 1 Pig Development Department, Teagasc Animal and Grassland Research and Innovation Centre, Moorepark, Fermoy, Co. Cork P61 C996 Ireland; 2 School of Veterinary Medicine, University College Dublin, Belfield, Dublin, D04 W6F6 Ireland; 3 Department of Animal Biosciences, University of Guelph, Guelph, N1G 2W1 Canada

**Keywords:** Health, Injurious behaviour, Pigs, Production stages, Welfare assessment

## Abstract

**Background:**

Knowledge on the most prevalent welfare problems for pigs in different production stages is required to improve herd management plans. Thirty-one farrow-to-finish pig farms were visited between July and November 2015 to assess the welfare of pigs using the multicriteria approach of the Welfare Quality® protocol. On each farm, 6 pens were selected using proportionate stratified sampling in the first weaner (S1, 4 to 8 wks), second weaner (S2, 8 to 13 wks) and finisher stage (S3, 13 to 23 wks), excluding hospital pens. Each pen was observed for 10 min and the number of pigs affected by different welfare outcomes was recorded. The percentage of pigs affected was calculated and ranked to identify the most prevalent outcomes within each production stage. Differences between production stages were analysed using generalised linear mixed models for binomial data with pen within stage and farm as a random effect.

**Results:**

Tail and ear lesions showed the highest prevalence; however, large variation was observed between farms. In S1 the most prevalent welfare outcomes (presented as median prevalence) were poor body condition (4.4%), lethargic pigs (1.5%), scouring (20.3% of pens) and huddling (3.7%). In S2 and S3 outcomes related to injurious behaviour (tail lesions: 5.9% [S2] and 10.5% [S3], ear lesions: 9.1% [S2] and 3.3% [S3], and flank lesions: 0.4% [S2] and 1.3% [S3]), lameness (0.8% [S2] and 1.1% [S3]), bursitis (3.9% [S2] and 7.5% [S3]) and hernias (1.6% [S2] and 1.8% [S3]) were more prevalent.

**Conclusions:**

A large variation was observed for the recorded welfare outcomes corresponding to the different challenges pigs experience during the different stages of production on commercial pig farms. The prevalence of pigs affected by lesions caused by injurious behavior is a cause for concern and requires a collaborative approach to identify appropriate intervention strategies. This information could be used to further investigate appropriate benchmark values for different welfare outcomes that would assist the pig industry to develop appropriate health and welfare management plans to minimise welfare problems. At herd level such plans should include information on aspects of intervention, treatment, and the management of hospital pens as well as euthanasia.

## Background

Recent performance figures for Irish pig herds from the Teagasc eProfit Monitoring system show a 10.6% piglet, 2.7% weaner and 2.4% finisher mortality rate, respectively [1]. However, reasons for mortality are not recorded in this system and they do not reflect the fact that pigs suffer health and performance setbacks such that they take longer to reach slaughter. A recent study found that pigs that were delayed (or ‘held back’) once or several times during production had lower carcass weights and higher odds of pericarditis and lameness at time of slaughter, indicating associations with welfare and/or (sub-)clinical disease challenges [2]. Such challenges are reflected in animal based outcome measures like those recorded under standardised protocols such as Welfare Quality® [3, 4]. This and similar protocols were used in several studies investigating the prevalence of various welfare outcomes in pigs at different stages of production [5–11]. The current study utilised a modified version of the Welfare Quality® protocol to determine the prevalence of welfare problems that occur between weaning and slaughter and how their occurrence differ between production stages (i.e. first and second weaner stage, and finisher stage) in 31 pig farms for the first time in Ireland.

## Methods

Farrow-to-finish pig farms were selected based on their participation in the Teagasc eProfit Monitor system and on the location of the abattoir they supplied as part of a larger study [12]. A total of 44 farrow-to-finish pig farms were contacted and 31 farms agreed to participate in the study (70.5%) representing approximately 12% of pig farms in Ireland. Farms were visited once (July to November 2015) and a cross-sectional welfare assessment of pigs in the first weaner (S1, 4 to 8 wks), second weaner (S2, 8 to 13 wks) and finisher stage (S3, 13 to 23 wks) was conducted. In brief, each farm visit lasted one day with producers being asked to provide an overview of farm lay-out and general aspects of management and production (e.g. mixing practices upon entering the production stage). Thereafter, six pens in each stage were selected using proportionate stratified sampling. The number of selected pens was based on similar previous cross-sectional studies and feasibility [4, 13–15]. Hospital pens and pens of pigs that entered a production stage within the previous 24 h were excluded to ensure that pens were representative of the general pig population on the farm. The number of pens selected in different houses on the farm was balanced according to the number of pigs in each house. All assessments were conducted by a trained observer from outside the pen in a similar manner to how a stockperson performs daily inspections of pens of pigs. The pigs in each pen were counted and observed for a 10 min period during which time different welfare outcomes (adapted from [4, 16–20]) were recorded (Table 1).

**Table 1:** Animal based welfare outcomes recorded during welfare assessment on 31 farms in the Republic of Ireland

Outcome	Description
Poor body condition	Lean pig with spine, hip and pin bones visible
Bursitis	Presence of inflamed bursae (tangerine-sized or larger) on limb(s)
Manure on the body^a^	More than half of pigs in the pen with > 50% of their body soiled
Shivering	Slow and irregular vibration of body or parts thereof
Panting	Breathing rapidly in short gasps carried out with the mouth
Huddling^b^	Pig lying with more than half of its body in contact with another pig
Lameness	Minimal weight-bearing on the affected limb or inability to move
Skin lesions	Presence of many large and/or deep skin lesions (i.e. more than 1/3 of the body covered with lesions)
Tail lesions	Moderate: evidence of lesions but no blood visible
	Severe: bloody, swollen and/or amputated tail
Ear lesions	Moderate: evidence of lesions but no blood visible
	Severe: bloody, swollen and/or amputated ear
Flank lesions	Moderate: evidence of lesion but no blood visible
	Severe: bloody, open wound
Lethargic	Pig appears generally listless/lethargic
Scouring^a^	Visible signs of liquid faeces on the floor in the pen
Coughing^c^	Frequency of coughs
Sneezing^c^	Frequency of sneezes
Pumping	Labored breathing; easily visible rising/falling of chest with each breath
Twisted snouts	Characteristic of atrophic rhinitis, deformity of the snout
Hernia and rupture	Hernia (umbilical/scrotal)/rupture present (i.e. larger than a fist)
Rectal prolapse	Internal tissue extrudes from the rectum
Skin condition	Skin is inflamed, discolored or spotted

^a^Recorded on pen level only (i.e. present/absent)

^b^Expressed as proportion of resting pigs

^c^Frequency per pig during 5 min observation. All other measures are assessed by recording the percentage of pigs affected in a pen

The number of pigs with poor body condition, bursitis, shivering, panting, huddling, lameness, skin lesions, tail lesions, ear lesions, flank lesions, lethargic behaviour, pumping, twisted snouts, hernias and rupture, rectal prolapse and skin conditions were recorded (Table 1). The severity of some types of injuries was assessed. Tail lesions were scored as either the presence of moderate or severe lesions [19, 21]. Similarly, the presence of moderate and severe ear and flank lesions was assessed on both sides of the body [17]. Scabbed over lesions were generally recorded as moderate lesions, however if the tail or ear was partly bitten off it was considered as severe. Hereafter, when referring to tail, ear, or flank lesions the total prevalence (moderate plus severe forms) is given unless otherwise indicated. Skin lesions which were caused by poor pen maintenance (i.e. clear, straight lesions) were not recorded. Either side of the body was assessed to detect the presence of lesions as per the Welfare Quality protocol [4]. Pigs that were observed to lie down during the entire observation period were encouraged to stand up (e.g. the observer entered the pen if necessary) to be able to accurately assess if these animals were lame.

Pig dirtiness was assessed at pen level and a pen was considered dirty when more than half of the pigs in the pen had > 50% of their body soiled [4]. The presence of scouring was recorded at pen level and assessed based on visible and fresh faeces on the floor in the pen [4]. A second observer recorded the frequency of coughs and sneezes per pen during the first 5 min. of the observation.

### Statistical analysis

The majority of the animal-based outcomes were recorded as number of pigs affected per pen and consequently expressed as the percentage of pigs affected per farm. For the pen level outcomes (‘manure on the body’ and ‘scouring’) the proportion of pens affected per farm was calculated. Coughing and sneezing were expressed as frequency per pig during the 5 min observation period. The median and interquartile range (IQR) of the prevalence of the different welfare outcomes were calculated for each of the production stages and ranked to identify the most prevalent outcomes within each production stage. We identified the number of farms where at least one animal (or pen in case of pen level outcomes) was affected by the welfare issue to see whether certain outcomes might be highly prevalent but only on a small number of farms or vice versa. Due to their low prevalence shivering, panting, pumping, twisted snout, rectal prolapse and skin condition were not analysed further.

All statistical procedures were conducted using SAS v9.3 (SAS Inst. Inc., Cary, NC). Alpha level for determination of significance and tendencies were 0.05 and 0.10 respectively. Spearman correlations were calculated to investigate possible associations between the different welfare outcomes on a farm level. Data were expressed as the number of animals affected out of the total of animals per pen. The effect of production stage on the prevalence of each separate welfare outcome was analysed using generalised linear mixed models for binomial data (PROC GLIMMIX) as per Temple et al. [9] using a random effect for pen within stage and farm. Similar models were created for the presence of dirtiness and scouring in pens. The frequency of coughing and sneezing was analysed using a gamma distribution.

## Results

### Description of study farms

The average size of participating farms was 751 ± 103.0 sows (range 111–2396). A total of 554 pens were observed accounting for a total of 17,414 pigs. On average, pigs entered S1 at 4 weeks, S2 at 8 weeks and S3 at 13 weeks of age. Average group size in S1 was 40.3 ± 1.71 pigs, 30.1 ± 0.92 pigs in S2 and 24.1 ± 0.82 in S3. Most pigs were kept in mixed-sex groups (female and entire males, 75.8% of pens) rather than all-male (entire males only, 13.0%) or all-female pens (females only, 11.2%). Nearly all pens had fully slatted flooring (91.9%) and 7.5% had partial slatted flooring. The most common flooring materials were plastic (44.2%, mostly in S1 [98%]) and concrete (55.8%, mostly in S2 [64%] and S3 [100%]). Pigs were tail docked on all farms.

### Prevalence of welfare outcomes on farm

In general, large variation was observed between farms for all welfare outcomes. Tail lesions were the most prevalent outcome recorded (Table 2). Correlations between the most common welfare outcomes are given in Table 3, showing that the prevalence of tail lesions and the prevalence of bursitis were highly correlated. Moderate correlations were found for poor body condition and lethargic pigs, huddling and coughing, and skin and flank lesions.

**Table 2:** Number and percentage (%) of farms with at least one pig affected by welfare outcomes including the median prevalence and interquartile range (IQR) of pigs affected per farm (%) of each welfare outcome observed on 31 Irish pig farms

	Farms		Prevalence	
			Median	IQR
	n	%	%	%
*Hunger*				
Poor body condition	31	100	2.47	1.85–3.48
*Comfort around resting*				
Bursitis	31	100	4.80	3.35–6.18
Manure on the body^a^	27	87.1	0.11	0.06–0.13
*Thermal comfort*				
Shivering	2	6.5	0.00	0.00–0.00
Panting	3	9.7	0.00	0.00–0.00
Huddling	23	74.2	1.32	0.00–2.50
*Injuries*				
Lameness	30	96.8	0.82	0.56–1.15
Skin lesions	31	100	4.77	3.11–7.09
Tail lesions (overall)	31	100	7.57	5.59–8.98
Moderate lesions	31	100	5.87	3.93–8.45
Severe lesions	25	80.6	0.83	0.08–1.78
Ear lesions (overall)	31	100	6.97	3.51–17.1
Moderate lesions	31	100	1.52	0.99–2.83
Severe lesions	31	100	5.42	2.96–14.93
Flank lesions (overall)	23	74.2	0.83	0.00–1.97
Moderate lesions	12	38.7	0.00	0.00–0.28
Severe lesions	22	71.0	0.69	0.00–1.83
*Disease*				
Lethargic	28	90.3	0.71	0.33–1.29
Scouring^a^	28	90.3	0.11	0.06–0.17
Coughing^b^	30	96.8	0.02	0.01–0.03
Sneezing^b^	31	100	0.17	0.13–0.24
Pumping	10	32.3	0.0	0.00–0.26
Twisted snout	0	0.0	0.0	0.00–0.00
Hernia	31	100	1.36	1.00–1.91
Rectal prolapse	2	6.5	0.0	0.00–0.00
Skin condition	11	35.5	0.0	0.00–0.29

^a^Average proportion of pens

^b^Frequency per pig during 5 min observation

**Table 3:** Spearman correlations between prevalence of the most common welfare outcomes and the frequency of coughing and sneezing on farm

	1	2	3	4	5	6	7	8	9	10	11
PBC^a^, 1											
Bursitis, 2	ns										
Huddling, 3	ns	ns									
Lameness, 4	ns	ns	−0.38^*^								
Skin lesions, 5	ns	ns	ns	ns							
Tail lesions, 6	ns	0.68^***^	ns	ns	ns						
Ear lesions, 7	ns	ns	ns	ns	ns	ns					
Flank lesions, 8	ns	ns	ns	−0.41^*^	0.44^*^	ns	ns				
Coughing, 9	ns	ns	0.44^*^	ns	ns	ns	ns	ns			
Sneezing, 10	ns	ns	ns	0.39^*^	ns	ns	ns	−0.44^*^	ns		
Hernia, 11	ns	ns	ns	ns	ns	ns	ns	ns	ns	ns	
Lethargic, 12	0.43^*^	ns	ns	ns	ns	ns	ns	ns	ns	ns	ns

^a^Poor body condition (PBC)

^*^P < 0.05; ^***^ *P* < 0.001

### Differences in prevalence of welfare outcomes between production stages

Prevalence of the different welfare outcomes in each production stage is shown in Table 4.

**Table 4:** Percentage (%) of farms with at least one pig affected by welfare outcomes including the median prevalence and interquartile range (IQR) of pigs affected per farm (%) of each welfare outcome observed in first weaner stage (S1), second weaner stage (S2) and finisher stage (S3) on 31 Irish pig farms

	S1		S2		S3	
	Farms %	Prevalence Median (IQR [%])	Farms %	Prevalence Median (IQR [%])	Farms %	Prevalence Median (IQR [%])
*Absence of hunger*						
Poor body condition	100	4.4 (3.53–7.06)	100	1.6 (1.28–2.42)	64.5	0.9 (0.00–1.63)
*Comfort at resting*						
Bursitis	83.9	1.0 (0.56–2.11)	100	3.9 (2.73–6.24)	100	7.5 (4.87–11.60)
*Thermal comfort*						
Shivering	3.2	0.0 (0.00–0.00)	3.2	0.0 (0.00–0.00)	0.0	0.0 (0.00–0.00)
Panting	0.0	0.0 (0.00–0.00)	3.2	0.0 (0.00–0.00)	6.5	0.0 (0.00–0.00)
Huddling	64.5	3.7 (0.00–7.33)	25.8	0.0 (0.00–0.62)	6.5	0.0 (0.00–0.00)
*Injuries*						
Lameness	48.4	0.0 (0.00–0.44)	74.2	0.8 (0.00–1.59)	74.2	1.1 (0.00–1.92)
Skin lesions	100	3.7 (1.85–8.62)	90.3	4.4 (1.86–6.27)	96.8	4.5 (3.45–7.34)
Tail lesions (overall)	100	2.8 (2.01–6.96)	100	5.9 (04.13–7.72)	100	10.5 (8.39–13.23)
Moderate lesions	100	2.6 (2.02–5.92)	100	5.3 (3.64–6.55)	100	8.8 (6.39–11.63)
Severe lesions	22.6	0.0 (0.00–0.00)	45.2	0.0 (0.00–1.04)	64.5	0.9 (0.00–3.30)
Ear lesions (overall)	96.8	7.6 (2.58–13.23)	93.5	9.1 (2.64–26.38)	87.1	3.3 (1.76–14.33)
Moderate lesions	93.5	2.7 (1.14–5.30)	83.9	1.3 (0.48–3.18)	54.8	0.6 (0.00–1.55)
Severe lesions	87.1	4.2 (0.48–7.11)	87.1	7.1 (1.84–25.11)	80.6	2.9 (0.93–9.71)
Flank lesions (overall)	16.1	0.0 (0.00–0.00)	51.6	0.4 (0.00–0.90)	71.0	1.3 (0.00–3.51)
Moderate lesions	3.2	0.0 (0.00–0.00)	19.4	0.0 (0.00–0.00)	29.0	0.0 (0.00–0.44)
Severe lesions	12.9	0.0 (0.00–0.00)	45.2	0.0 (0.00–0.76)	67.7	1.3 (0.00–2.78)
*Disease*						
Lethargic	90.3	1.5 (0.86–2.45)	41.9	0.0 (0.00–0.68)	38.7	0.0 (0.00–0.69)
Coughing^a^	87.9	0.0 (0.0–0.04)	74.2	0.0 (0.00–0.03)	61.3	0.0 (0.00–0.02)
Sneezing^a^	100	0.4 (0.13–0.57)	100	0.2 (0.09–0.20)	96.8	0.1 (0.02–0.09)
Hernia	80.6	1.0 (0.28–1.24)	87.1	1.6 (0.79–2.38)	87.1	1.8 (0.98–3.49)

^a^Frequency per pig during 5 min observation

Differences were found between stages for all welfare outcomes except for the prevalence of skin lesions (Table 5). Pigs in S1 had an increased risk of poor body condition, lethargic behaviour and huddling compared to S2 and S3. The frequency of coughing tended to be lower in S1 (0.04 ± 0.007 coughs/pig) compared to S2 (0.07 ± 0.008 coughs/pig, *P* = 0.05) and was lower compared to S3 (0.09 ± 0.013 coughs/pig, *P* < 0.01). No difference was found in frequency of coughing between S2 and S3 (*P* > 0.05). In contrast, frequency of sneezing decreased with age (S1: 0.34 ± 0.038, S2: 0.21 ± 0.018, S3: 0.10 ± 0.011 sneezes/pig, *P* < 0.001). The percentage of pens in which signs of scouring were observed was higher in S1 (20.3 ± 2.99%) compared to S2 (8.1 ± 2.00%) and S3 (7.0 ± 1.87%, *P* < 0.001). Pens where the majority of pigs had > 50% of their body soiled were more frequently observed in S2 (11.3 ± 2.33%) and S3 (16.7 ± 2.74%) compared to S1 (0.6 ± 0.55%, *P* < 0.01).

**Table 5:** Odds ratio and 95% confidence interval (CI) for each animal-based welfare outcome associated with production stage (first weaner stage [S1], second weaner stage [S2], and finisher stage [S3])

	S1 vs S2		S1 vs S3		S2 vs S3	
	OR	95% CI	OR	95% CI	OR	95% CI
*Absence of hunger*						
Poor body condition	0.4^*^	0.32–0.52	0.2^*^	0.15–0.30	0.5^*^	0.37–0.76
*Comfort around resting*						
Bursitis	3.7^*^	2.83–4.77	6.6^*^	5.16–8.55	1.8^*^	1.50–2.18
*Thermal comfort*						
Huddling	0.2^*^	0.12–0.39	0.1^*^	0.02–0.18	0.2^*^	0.07–0.89
*Injuries*						
Lameness	3.3^*^	1.97–5.56	4.6^*^	2.75–7.66	1.4	0.94–2.01
Skin lesions	1.2	0.89–1.58	1.4	1.01–1.80	1.1	0.85–1.52
Tail lesions (overall)	1.5^*^	1.14–1.85	2.8^*^	2.19–3.52	1.9^*^	1.52–2.41
Moderate lesions	1.5^*^	1.21–1.91	2.6^*^	2.12–3.30	1.7^*^	1.41–2.15
Severe lesions	1.8	0.90–3.71	4.7^*^	2.46–9.07	2.6^*^	1.43–4.68
Ear lesions (overall)	1.5	0.98–2.24	0.7	0.44–1.05	0.5^*^	0.30–0.71
Moderate lesions	0.6^*^	0.44–0.85	0.3^*^	0.18–0.42	0.4^*^	0.28–0.70
Severe lesions	2.5^*^	1.59–4.02	1.2	0.76–2.01	0.5^*^	0.31–0.78
Flank lesions (overall)	5.2^*^	2.39–11.24	9.6^*^	4.49–20.34	1.8	1.08–3.14
Moderate lesions^a^	–	–	–	–	–	–
Severe lesions	4.8^*^	2.12–10.81	9.1^*^	4.14–20.12	1.9	1.09–3.34
*Disease*						
Lethargic	0.3^*^	0.19–0.46	0.2^*^	0.14–0.41	0.8	0.44–1.54
Hernia	1.8^*^	1.25–2.47	2.3^*^	1.68–3.29	1.3	0.98–1.83

^a^Model did not converge

^*^Significantly different from the reference category; *P* < 0.05

The first mentioned production stage is the reference category within each column

An increase in the prevalence of bursitis across production stages was found. A similar increase was observed for the prevalence of lameness and hernias; however, no differences were observed between S2 and S3 for these outcomes. Tail and flank lesions showed a similar pattern, with higher prevalence rates as the pigs progressed through the production stages. The prevalence of severe tail lesions was higher in S3 compared to S1 and S2. The highest prevalence of ear lesions was found in S2, especially in the case of severe ear lesions.

## Discussion

This study provides results of a welfare assessment of pigs at different stages of the production cycle on 31 Irish farms. Farms were selected based on the criteria that they were farrow-to-finish farms, kept records in the Teagasc eProfit Monitor, and supplied pigs to local abattoirs as part of another study [12]. Results from such convenience sample where farmers volunteered to participate should be interpreted with caution; however, these farms represent nearly 25% of all herds in the Teagasc eProfit Monitor which contains records for over 65% of the national sow herd [1]. Additionally, sow herd size and production figures of the 31 farms were similar to the average Irish production figures based on the Teagasc eProfit Monitor [1].

Some limitations regarding the methodology of this study should be discussed. It is important to keep in mind that this cross-sectional welfare assessment was a snapshot of the welfare of pigs on farm. Hospital pens were excluded as this would give a skewed image of the welfare of pigs on the farm. Six pens were selected within each production stage and care was taken to select pens proportionately to the number of pigs in different houses. This maximised the number of pens and consequently pigs observed in each production stage in a way that was feasible in the time frame for the assessment. At the same time, pigs were observed from outside the pen further reducing the time needed for the assessment rather than more in-depth inspections of individual pigs. This approach of assessing pigs from outside the pen has been used in previous studies [10, 17, 21] and is more similar to how a stockperson would inspect pigs. Pigs affected by the different welfare outcomes were often easy to recognize once observed; however, with varying group sizes it is possible that some welfare outcomes were missed. Nonetheless, since the methodology was the same for each farm, it is assumed that this study provides a valuable estimate of the prevalence of different welfare outcomes in three production stages on Irish farms and power calculations revealed a good power (> 0.8) for testing for differences between the stages for the welfare outcomes considered. Additionally, we presented the number of farms that had pigs affected by the different welfare outcomes similar to Smulders et al. [21] to gain an understanding of whether welfare outcomes were observed on a large proportion of farms. This was a low threshold value for farms to be included and as such is an overestimation. Investigation into appropriate thresholds for the different welfare outcomes to benchmark farms and further validation of the methodology is necessary if it is to be extended for use in pig welfare assurance schemes. Such work was outside of the scope of this descriptive study.

Although the median prevalence of many of the welfare outcomes was low, the conditions were observed on a high proportion of farms. However, it should be noted that only one animal needed to be affected (or one pen in the case of pig dirtiness and scouring) for the farm to be considered as positive for the particular welfare outcome. Encouragingly, there were also farms where no cases of manure on the body, shivering, panting, lameness, severe tail lesions, flank lesions, lethargic pigs, scouring, coughing, pumping, twisted snout, rectal prolapse or skin conditions were found, though hospital pens were excluded and so results should be interpreted with caution. Future research should investigate how these farms differed in terms of management from farms where high proportions of animals were affected with some of the aforementioned conditions.

The low prevalence of, for example, rectal prolapses could reflect that this condition is relatively easily detected and therefore more likely to be appropriately managed on farms such that pigs are immediately removed to a hospital pen or euthanised upon detection [22]. In contrast, the prevalence of pigs affected by lesions caused by injurious behaviour could suggest a lack of an effective straightforward strategy to address conditions of a multifactorial nature [3]. The prevalence of lesions caused by injurious behaviour may also make it logistically problematic to hospitalise affected pigs, as large numbers of pigs could be affected at the same time. This could lead to the normalisation of a common injury i.e. “when bad becomes normal” [23], and tolerance towards certain welfare issues such as tail lesions [24]. In addition, some producers are reluctant to use hospital pens or use different criteria when deciding if a pig is ‘sick enough’ to be moved to a hospital pen [25]. Research into the attitudes of pig producers regarding use of hospital pens and their tolerance for certain welfare outcomes is needed to devise proper management strategies.

Several studies assessed the differences in prevalence of welfare outcomes between different age/weight groups of pigs [7, 9, 26]; and it should be noted that similar trends were observed across countries with large variation in the prevalence of welfare outcomes [5, 8, 10, 26]. The following sections discuss the results of the welfare assessment according to the welfare criteria defined by the Welfare Quality® [4].

### Absence of prolonged hunger

Pigs in poor body condition were predominantly seen in S1. Weaning is associated with many stressors for pigs, and has a detrimental impact on feed intake, growth and health [27, 28]. A moderate correlation was found between poor body condition and lethargic pigs. In accordance with other studies the prevalence of pigs in poor body condition decreased with each successive production stage [9, 26].

### Comfort around resting

Pens with soiled pigs were more often observed in S2 and S3 compared to S1. Pigs tend to become increasingly dirty as they age [8, 26], probably due to changes in housing such as flooring characteristics and stocking density or increased time spent lying down [29, 30].

Bursitis was one of the most prevalent outcomes observed and is likely underreported in this study as the methodology meant that only severe cases (larger than a tangerine) were recorded. Our findings were similar to other reports for severe bursitis [8, 13]. Bursitis was more prevalent in S2 and S3 pens compared to S1 pens. Age is a risk factor for bursitis [9, 13, 14, 26] attributable to increased pressure on the joints due to higher body weight. In addition, older pigs are likely to be housed on concrete floors (as was observed in this study), which contributes to the development of bursitis [14].

### Thermal comfort

Signs of thermal discomfort were rarely observed during the study. Huddling decreased from S1 to S3 which is similar to the trend observed by Temple et al. [9], which could reflect different requirements for thermal comfort associated with age with younger pigs requiring higher temperatures. Huddling can also be a sign of lethargic behaviour [31], which could in part explain the high prevalence in S1.

### Absence of injuries

Only pigs showing severe lameness (i.e. minimal weight-bearing or inability to move) were detectable from outside the pen. The prevalence of such cases in finisher pigs was similar to those found in other studies [7, 11, 32]. The higher prevalence of lameness observed in S2 and S3 compared to S1 could be related to higher body weights and concrete flooring which are risk factors for lameness [13, 15, 33].

The prevalence of injuries such as skin or tail lesions were higher in this study compared to those reported by a large scale study in the UK [11]; however, the latter study used farms under the Red Tractor Assurance scheme which could help explain this difference. Skin lesions are indicators of aggressive behaviour and were observed at a relatively similar prevalence in each production stage. Tail and ear lesions, and to lesser extent flank lesions, were among the most prevalent welfare outcomes recorded which is in agreement with Petersen et al. [7]. However, the majority of tail lesions observed were considered moderate lesions, while ear and flank lesions were more often seen in their severe form. The prevalence of tail lesions increased as the production stages progressed. Other studies report a similar prevalence for tail lesions in S2 and S3 [5], and severe tail lesions in S3 [26]. Flank lesions were observed at all stages albeit at a higher prevalence in S2 and S3. Flank and ear lesions likely share the same pathogenesis associated with infection with *S hyicus, S aureus* or *Treponema spp.* which can occur after damage to the flanks or ears of weaned pigs (e.g. by ear or flank biting [34, 35]). The study could not determine whether ear lesions were caused by ear biting or ear necrosis; however, it is likely that both conditions played a role in the high prevalence of ear lesions observed. This study showed a high prevalence of ear lesions in S2 followed by a reduction in S3 in agreement with other studies [5, 7].

### Absence of disease

The Welfare Quality® protocol assesses the absence of disease by looking at several separate outcomes [4]; however, we included an additional category; lethargic pigs. As mentioned previously, weaning is associated with stress-related morbidity [27, 28] and therefore it is not surprising that the prevalence of lethargic pigs was highest in S1. Pigs likely recovered from these initial challenges as shown by the low prevalence in the subsequent stages. The prevalence of scouring in this study was similar to that observed on 30 Spanish pig farms [8]. Scouring is often observed post-weaning due to changes in feed [27, 28], explaining the higher proportion of pens affected in S1 compared to S2 and S3. The frequency of coughs and sneezes was determined per pig, while other studies typically report on the percentage of pigs affected making comparison difficult [5, 8]. Coughing tended to become more frequent from S2 onward, which reflects that respiratory disease is more common in older pigs [4].

Hernias were observed at a higher prevalence in S2 and S3 compared to S1. The prevalence is higher than reported in finisher pigs in Denmark [7], but could partly be explained by the fact that they only included umbilical hernias whilst we recorded both scrotal and umbilical hernias. It is possible that hernias are more commonly observed from S2 onwards because growth of the pigs in combination with increased weight of the abdominal content leads to larger hernias [22]. Higher mortality rates and reduced growth rates are observed in pigs with umbilical hernias [36] suggesting that, apart from welfare concerns, it may also be more profitable to identify and euthanise these pigs.

## Conclusions

This study provides an overview of the prevalence of welfare outcomes on Irish pig farms, which are similar to those reported by studies from other countries. A large variation was observed for the recorded welfare outcomes corresponding to the different challenges pigs experience throughout the different stages of production. The prevalence of pigs affected by lesions caused by injurious behaviour is a cause for concern and requires a collaborative approach to identify appropriate intervention strategies. The findings from this study could be used to further investigate appropriate benchmark values for different welfare outcomes that would assist the pig industry to develop appropriate health and welfare management plans to minimise welfare problems. At herd level such plans should include information on aspects of intervention, treatment, and the management of hospital pens as well as euthanasia.

## References

[1] Teagasc. The National Pig Herd Performance Report - 2015. Fermoy: Pig Development Department, Teagasc Animal and Grassland Research and Innovation Centre. p. 2016.

[2] Calderón Díaz JA, Diana A, Boyle LA, Leonard FC, McElroy M, McGettrick S, Moriarty J, Manzanilla EG. Delaying pigs from the normal production flow is associated with health problems and poorer performance. Porcine Health Manag. 2017;3:13. 10.1186/s40813-017-0061-6. 10.1186/s40813-017-0061-6 PMC5497354 28690865

[3] Scientific Opinion EFSA (2012). On the use of animal-based measures to assess welfare in pigs. The EFSA Journal.

[4] Welfare Quality (2009). Welfare Quality® assessment protocol for pigs (sows and piglets, Growing and finishing pigs).

[5] Goossens X, Sobry L, Ödberg F, Tuyttens F, Maes D, de Smet S, Nevens F, Opsomer G, Lommelen F, Geers RA (2008). Population-based on-farm evaluation protocol for comparing the welfare of pigs between farms. Anim Welf.

[6] Munsterhjelm C, Heinonen M, Valros A (2015). Application of the welfare quality® animal welfare assessment system in Finnish pig production, part I: identification of principal components. Anim Welf.

[7] Petersen HH, Nielsen E, Hassing A, Ersbøll AK, Nielsen JP (2008). Prevalence of clinical signs of disease in Danish finisher pigs. Vet Rec.

[8] Temple D, Dalmau A, Ruiz de la Torre JL, Manteca X, Velarde A (2011). Application of the welfare quality® protocol to assess growing pigs kept under intensive conditions in Spain. J Vet Behav Clin Appl Res.

[9] Temple D, Courboulay V, Manteca X, Velarde A, Dalmau A (2012). The welfare of growing pigs in five different production systems: assessment of feeding and housing. Animal.

[10] Whay HR, Leeb C, Main DCJ, Green LE, Webster AJF (2007). Preliminary assessment of finishing pig welfare using animal-based measurements. Anim Welf.

[11] Pandolfi F, Stoddart K, Wainwright N, Kyriazakis I, Edwards SA (2017). The ‘real welfare’ scheme: benchmarking welfare outcomes for commercially farmed pigs. Animal.

[12] van Staaveren N, Doyle B, Manzanilla EG, Calderón Díaz JA, Hanlon A, Boyle L (2017). Validation of carcass lesions as indicators for on-farm health and welfare of pigs. J Anim Sci.

[13] Quinn AJ (2014). Limb health in pigs: the prevalence and risk factors for lameness, limb lesions and claw lesions in pigs, and the influence of gilt nutrition on indicators of limb health. PhD thesis. University of Warwick.

[14] Gillman CE, KilBride AL, Ossent P, Green LE (2008). A cross-sectional study of the prevalence and associated risk factors for bursitis in weaner, grower and finisher pigs from 93 commercial farms in England. Prev Vet Med.

[15] KilBride AL, Gillman CE, Green LEA (2009). Cross-sectional study of the prevalence of lameness in finishing pigs, gilts and pregnant sows and associations with limb lesions and floor types on commercial farms in England. Anim Welf.

[16] Björklund L. Effects of rearing boars and gilts in mixed- or single-sex groups and split-marketing on behaviour, welfare and performance. Linköping: Linköpings universitet - institute of. Technology. 2005;

[17] Diana A, Manzanilla EG, Calderón Díaz JA, Leonard FC, Boyle LA. Do weaner pigs need in-feed antibiotics to ensure good health and welfare? PLoS ONE. 2018;13(2):e0193505. 10.1371/journal.pone.0193505. 10.1371/journal.pone.0185622 PMC5628837 28982114

[18] Harley S, More SJ, O'Connell NE, Hanlon A, Teixeira D, Boyle L (2012). Evaluating the prevalence of tail biting and carcase condemnations in slaughter pigs in the republic and Northern Ireland, and the potential of abattoir meat inspection as a welfare surveillance tool. Vet Rec..

[19] Kritas SK, Morrison RB (2007). Relationships between tail biting in pigs and disease lesions and condemnations at slaughter. Vet Rec..

[20] Main DCJ, Clegg J, Spatz A, Green LE (2000). Repeatabilty of a lameness scoring system for finishing pigs. Vet Rec..

[21] Smulders D, Hautekiet V, Verbeke G, Geers R (2008). Tail and ear biting lesions in pigs: an epidemiological study. Anim Welf.

[22] St. Jean G, Anderson DE. Anesthesia and Surgical Procedures in Swine. In: Straw BE, Zimmerman JJ, D'Allaire S, Taylor DJ, editors. Disease of Swine - 9th edition. Ames: Blackwell Publishing; 2006. p. 119–141.

[23] Grandin T (2003). Transferring results of behavioral research to industry to improve animal welfare on the farm, ranch and the slaughter plant. Appl Anim Behav Sci.

[24] Devitt C, Boyle L, Teixeira DL, O’Connell NE, Hawe M, Hanlon A (2016). Pig producer perspectives on the use of meat inspection as an animal health and welfare diagnostic tool in the Republic of Ireland and Northern Ireland. Ir Vet J.

[25] Thomsen PT, Klottrup A, Steinmetz H, Herskin MS (2016). Attitudes of Danish pig farmers towards requirements for hospital pens. Res Vet Sci.

[26] Meyer-Hamme SEK, Lambertz C, Gauly M (2016). Does group size have an impact on welfare indicators in fattening pigs?. Animal.

[27] Campbell JM, Crenshaw JD, Polo J (2013). The biological stress of early weaned piglets. J Anim Sci Biotechnol.

[28] Lallès J-P, Bosi P, Smidt H, Stokes CR (2007). Weaning - a challenge to gut physiologists. Livest Sci.

[29] Aarnink AJA, Schrama JW, Heetkamp MJW, Stefanowska J, TTT H (2006). Temperature and body weight affect fouling of pig pens. J Anim Sci.

[30] Averós X, Brossard L, Dourmad JY, de Greef KH, Edge HL, Edwards SA, Meunier-Salaün MC (2010). Quantitative assessment of the effects of space allowance, group size and floor characteristics on the lying behaviour of growing-finishing pigs. Animal.

[31] Cook NJ, Chabot B, Lui T, Bench CJ, Schaefer AL (2015). Infrared thermography detects febrile and behavioural responses to vaccination of weaned piglets. Animal.

[32] Mullan S, Edwards SA, Butterworth A, Whay HR, Main DCJ (2009). Interdependence of welfare outcome measures and potential confounding factors on finishing pig farms. Appl Anim Behav Sci.

[33] Knage-Rasmussen KM, Houe H, Rousing T, Sørensen JT (2014). Herd- and sow-related risk factors for lameness in organic and conventional sow herds. Animal.

[34] Mirt D (1999). Lesions of so-called flank biting and necrotic ear syndrome in pigs. Vet Rec..

[35] Park J, Friendship RM, Poljak Z, DeLay J, Slavic D, Dewey CE (2013). An investigation of ear necrosis in pigs. Can Vet J.

[36] Straw B, Bates R, May G (2009). Anatomical abnormalities in a group of finishing pigs: prevalence and pig performance. J Swine Health Prod.

